# Lysosomal membrane permeabilization causes secretion of IL-1β in human vascular smooth muscle cells

**DOI:** 10.1007/s00011-018-1178-z

**Published:** 2018-08-22

**Authors:** Hiroaki Ono, Ryo Ohta, Yuri Kawasaki, Akira Niwa, Hidetoshi Takada, Tatsutoshi Nakahata, Shouichi Ohga, Megumu K. Saito

**Affiliations:** 1 Department of Clinical Application, Center for iPS Cell Research and Application, Kyoto University, Kyoto, 606-8507 Japan; 2 Department of Pediatrics, Graduate School of Medical Sciences, Kyushu University, Fukuoka, 812-8582 Japan; 3 Department of Perinatal and Pediatric Medicine, Graduate School of Medical Sciences, Kyushu University, Fukuoka, 812-8582 Japan; 4 Department of Child Health, Faculty of Medicine, University of Tsukuba, Ibaraki, 305-8575 Japan

**Keywords:** Inflammasome, Lysosomal membrane permeabilization (LMP), Human vascular smooth muscle cells (hVSMCs), NF-κB, Cathepsin B

## Abstract

**Objective:**

IL-1β secretion by the inflammasome is strictly controlled and requires two sequential signals: a priming signal and an activating signal. Lysosomal membrane permeabilization (LMP) plays a critical role in the regulation of NLRP3 inflammasome, and generally acts as an activating signal. However, the role of LMP controlling NLRP3 inflammasome activation in human vascular smooth muscle cells (hVSMCs) is not well defined.

**Methods:**

LMP was induced in hVSMCs by Leu-Leu-*O*-methyl ester. Cathepsin B was inhibited by CA-074 Me. Cytokine release, mRNA, and protein were quantified by enzyme-linked immunosorbent assay, quantitative PCR, and Western blot, respectively. NF-κB activity was analyzed by immunostaining of the NF-κB p65 nuclear translocation and using the dual-luciferase reporter assay system.

**Results:**

LMP had both priming and activating roles, causing an upregulation of proIL-1β and NLRP3 and the secretion of mature IL-1β from unprimed hVSMCs. LMP activated the canonical NF-κB pathway. The priming effect of LMP was inhibited by CA-074 Me, indicating an upstream role of cathepsin B.

**Conclusions:**

These data support a novel role of LMP as a single stimulus for the secretion of IL-1β from hVSMCs, implying the possibility that hVSMCs are an important initiator of the sterile inflammatory response caused by lysosomal disintegration.

## Introduction

Inflammation is a host response caused by the immune system against harmful stimulation. The inflammatory response should be tightly regulated, because an excessive inflammatory response can cause systemic inflammatory disease, while a poor inflammatory response can lead to a persistent or fatal infection of pathogens. The inflammasome is one of the most critical machineries for regulating the innate immune response. It initiates a proinflammatory response by recognizing damage-associated and pathogen-associated molecular patterns on its pattern recognition receptors (PRPs) [1]. The inflammasome is subcategorized according to the PRPs incorporated, and among them, the NLRP3 inflammasome is one of the most investigated for its involvement in various inflammatory disorders and conditions [2].

The NLRP3 inflammasome is a protein complex involved in the secretion of proinflammatory cytokines interleukin (IL)-1β and IL-18, and is typically composed of a PRP NLRP3, apoptosis-associated speck-like protein (ASC), and a protease caspase-1. The secretion of IL-1β requires two sequential signals: a priming signal and an activating signal [3]. The first priming signal, which is evoked by LPS and TNFα, primes the cells to upregulate the immature form of IL-1β (pro IL-1β) and some inflammasome components. Then, the secondary activating signal acts on the primed cell to assemble the inflammasome complex. For the NLRP3 inflammasome, there are a number of identified activating signals, such as extracellular ATP, potassium efflux, intracellular reactive oxygen species (ROS), and increased lysosomal membrane permeabilization (LMP). Activation of the NLRP3 inflammasome causes auto-cleavage of the pro-form of caspase-1, and the resulting activated caspase-1 then converts pro IL-1β into mature active IL-1β [4, 5]. Activation of the NLRP3 inflammasome also causes programmed cell death by pyroptosis or pyronecrosis. This two-step mechanism tightly controls the secretion of IL-1β and the activation of the NLRP3 inflammasome. Currently, there is no known cell type that secretes mature IL-1β with a single stimulation, except for human monocytes, which can produce mature IL-1β with a single LPS stimulation [6, 7].

Vascular smooth muscle cells (VSMCs) compose the majority of blood vessel walls at the vascular media, which locates at the outer layer of vascular endothelial cells. The main function of VSMCs is to adjust the diameter of the blood vessels in response to stimuli. The vascular media can also be the site of sterile inflammation, because immune cells infiltrate there [8]. Especially in the case of atherosclerosis, chronic inflammation is evoked by infiltrating macrophages that are activated by cholesterol crystals and oxidized low-density lipoproteins [9]. Since cholesterol crystals act as an activating signal for the NLRP3 inflammasome, activated macrophages produce large amounts of IL-1β while infiltrating the media [9, 10]. Interestingly, some human VSMCs (hVSMCs) express components of the NLRP3 inflammasome at the site of atherosclerosis, and hVSMCs themselves can contribute to local inflammation [11]. However, the role of hVSMCs on inflammasome-mediated vascular inflammation has not been fully elucidated.

Here, we found that a single signal, LMP, is enough to secrete IL-1β from unprimed hVSMCs. LMP is known as an important activating signal for the NLRP3 inflammasome, because activation of the NLPR3 inflammasome in various chronic inflammatory disorders, such as gout, atherosclerosis, and diabetes mellitus, takes place as a consequence of lysosomal disintegration [12]. On the other hand, in hVSMCs, in vitro experiments indicate that LMP can be both the priming and activating signal, making it sufficient for mature IL-1β secretion. These data highlight previously unknown roles in the inflammatory response by hVSMCs and provide insights into the pathophysiology of chronic vascular inflammation.

## Materials and methods

### Materials

Human aortic smooth muscle cells (HASMCs, C0075C), human coronary artery smooth muscle cells (HCASMCs, C0175C), and human pulmonary artery smooth muscle cells (HPASMCs, C0095C) were purchased from Thermo Fisher Scientific (Waltham, MA). Leu-Leu-*O*-methyl ester (LLME, L7393), L-3-trans-(propylcarbamyl)oxirane-2-carbonyl-l-isoleucyl-Lf-proline methyl ester (CA-074 Me, C5732), and ATP (A2383) were purchased from Sigma-Aldrich (St. Louis, MO). Caspase-1 inhibitor Ac-Tyr-Val-Ala-Asp-CHO (YVAD, 400010) was purchased from Merck (Darmstadt, Germany). Diphenyliodonium chloride (DPI, D2356) was purchased from Tokyo Chemical Industry (Tokyo, Japan). MG132 (1748) was purchased from R&D Systems (Minneapolis, MN). LPS from *Escherichia coli* K12 (tlrl-peklps) and MCC950 (inh-mcc) were purchased from InvivoGen (San Diego, CA). Nigericin (145-07263) and ionomycin (091-05833) were purchased from Wako Pure Chemical Industries (Osaka, Japan).

### Cell cultures

HVSMCs (HASMCs, HCASMCs, and HPASMCs) were cultured in Smooth Muscle Cell Growth Medium-2 (CC-3182; Lonza, Basel, Switzerland). Human pluripotent stem cell-derived immortalized monocytic cell lines (MLs) were cultured as previously described [13] in StemPro-34 serum-free medium (10639011; Thermo Fisher Scientific) containing 2 mM of l-glutamine and 50 ng/mL of M-CSF (216-MC; R&D Systems).

### Cytokine assays

HVSMCs were seeded into 12-well plates (353043; Corning Incorporated, Corning, NY) and cultured until 100% confluency. Cells were incubated with either 400 µL of culture medium (negative control) or LLME for the indicated time periods. In some experiments, inhibitors (YVAD, MCC950, 50 µM of CA-074 Me, 20 µM of DPI, or 10 µM of MG132) were added 1 h prior to the LLME stimulation. To prepare primed HASMCs, cells were stimulated with 100 ng/mL TNFα for 24 h. Primed or unprimed HASMCs were stimulated with 2.5 mM LLME, 5 µM ionomycin, or 1 µM nigericin for 24 h. MLs were stimulated with 1 µg/mL LPS for 24 h to prepare primed MLs. Primed or unprimed MLs adjusted to 1 × 10^6^ cells/mL were suspended in culture medium at the indicated concentration of LLME or 2.5 mM ATP for the indicated time periods. The supernatants were collected after centrifugation at 10,000×*g* for 5 min and stored at − 80 °C until assayed. Concentrations of cytokines were measured by LEGENDplex Multi-Analyte Flow Assay Kit (BioLegend, San Diego, CA) in accordance with the manufacturer’s instructions. Quantification of the cytokines was done with a BD LSRII Flow Cytometer (BD Biosciences, San Jose, CA). For stimulation, cells were treated with reagents for the indicated time periods.

### FITC dextran imaging

HASMCs were seeded onto multi-well glass-bottom dishes (D141400; Matsunami, Osaka, Japan), cultured with 0.5 mg/mL of FITC dextran (F0918; Tokyo Chemical Industry) for 1 h, and washed with PBS (−) twice. The cells were then incubated in culture medium with or without 2.5 mM LLME for 10 or 60 min. The cells were visualized with a FluoVIew10i confocal microscope (Olympus, Tokyo, Japan) and analyzed with ImageJ software (National Institutes of Health, Bethesda, MD).

### Immunostaining, NF-κB p65

HASMCs were seeded onto multi-well glass-bottom dishes. The nuclear translocation of NF-κB p65 subunit was evaluated as previously described [14]. After culture for the indicated time periods, cells were fixed with 4% paraformaldehyde in PBS (−) for 30 min. Permeabilization and blocking were performed with blocking buffer [blocking One (02952, Nacalai Tesque, Kyoto, Japan) with 0.1% Tween20 (9005-64-5; Santa Cruz Biotechnology, Dallas, TX)] for 30 min. Samples were then incubated with anti-NF-κB p65 rabbit mAb (8242S; Cell Signaling Technologies, Danvers, MA) in blocking buffer overnight at 4 °C and subsequently incubated with Alexa Fluor 488 goat anti-rabbit IgG (4412S; Cell Signaling Technologies) for 30 min. The nuclei were stained with 49-6-diamidino-2-phenylindole dihydrochloride (DAPI, 32670; Sigma-Aldrich) in PBS (−). The cells were visualized with a FluoVIew10i confocal microscope and analyzed with ImageJ software.

### Immunoblotting

Cell lysates were lysed in radioimmunoprecipitation buffer (188-02453; Wako Pure Chemical Industries) plus proteinase inhibitor cocktail (04080; Nacalai Tesque) and 1 µg/mL of 2-Mercaptoethanol (21417; Nacalai Tesque). After centrifugation at 15,000×*g* for 10 min at 4 °C, supernatants were collected. Culture supernatants were collected and concentrated by ultrafiltration using an Amicon Ultra device (UFC500324; Merck) with a 3 kDa molecular weight cutoff. Samples were boiled in laemmli sample buffer (1610737; Bio-Rad, Hercules, CA) with 100 mM dithiothreitol at 95 °C for 5 min. Proteins were then separated using SDS–PAGE and transferred to a polyvinylidene difluoride membrane. The membranes were blocked with PVDF Blocking Reagent for Can Get Signal (NKB101; Toyobo, Osaka, Japan) and immunoblotted using the following antibodies: mouse anti-NLRP3 (Cryo-2; AG-20B-0014-C100; Adipogen, San Diego, CA), rabbit anti-IL-1β (3866; Abcam, Cambridge, UK), rabbit anti-Caspase-1 (5125; Cell Signaling Technology), mouse anti-ASC (D086-3; Medical and Biological Laboratories, Nagoya, Japan), rabbit anti-IκBα (9242; Cell Signaling Technology), and HRP-conjugated rabbit anti-β-actin (5125; Cell Signaling Technology). The anti-IL-1β and anti-Caspase-1 antibodies recognize both the pro and mature forms of the proteins. HRP-labeled horse anti-mouse (7076) and HRP-labeled goat anti-rabbit (7074) were purchased from Cell Signaling Technology and used as secondary antibodies. Immunoreactive bands were visualized using SuperSignal Western Blot Enhancer (46640; Thermo Fisher) and detected with LAS4000 (Fujifilm, Tokyo, Japan).

### Cell viability assay

Cell number and viability were measured with a Countess Automated Cell Counter (Invitrogen, Carlsbad, CA).

### Quantitative PCR

RNA samples were prepared from the cells with RNeasy Mini Kit (74106; Qiagen, Hilden, Germany). RNA was then subjected to reverse transcription with PrimeScript RT Master Mix (RR036B; Takara Bio Inc, Otsu, Japan). All procedures were performed in accordance with the manufacturer’s instructions. Quantitative Polymerase chain reaction (qPCR) was performed on the Step One Plus Real-Time PCR System (Applied Biosystems, Foster City, CA). cDNA was subjected to qPCR, and SYBR Premix ExTaqII (RR390B; Takara Bio Inc) was used for the detection. Data were processed according to the ΔΔ cycle threshold method, and the relative quantities are shown. Forward and reverse primers were as follows: (1) *NLRP3*: 5′-ATGTGGGGGAGAATGCCTTG-3′, 5′-TTGTCTCCGAGAGTGTTGCC-3′; (2) *IL1B*: 5′-TCGCCAGTGAAATGATGGCT-3′, 5′-TGAAGCCCTTGCTGTAGTGG-3′; and (3) β-actin: 5′-ACAGAGCCTCGCCTTTGC-3′, 5′-CCACCATCACGCCCTGG-3′.

### NF-κB dual-luciferase assay

HASMCs were transfected with pGreenFire NF-κB transcription reporter lentivector (System Biosciences, Mountain View, CA) inserted with the Renilla gene under the CMV promoter as an internal control [14]. The NF-κB activity was then measured using the Dual-Luciferase Reporter Assay System (Promega, Madison, WI).

### Measurement of the intracellular ROS

To measure intracellular ROS, cells were incubated in medium with or without LLME for 1 h. CellROX Deep Red Reagent (C10422; Thermo Fisher) was added for the last 30 min at 37 °C. Cells were then collected and washed three times with PBS containing DAPI and assayed using BD LSRII Flow Cytometer. The data were analyzed with FlowJo software (TreeStar, Ashland, OR). Dead cells (DAPI+ cells) and debris were excluded by gating in the software. The mean fluorescence intensity of CellROX in each sample was regarded as the amount of ROS.

## Results

### Single LMP stimulation is enough to secrete mature IL-1β from unprimed hVSMCs

**Fig. 1:**
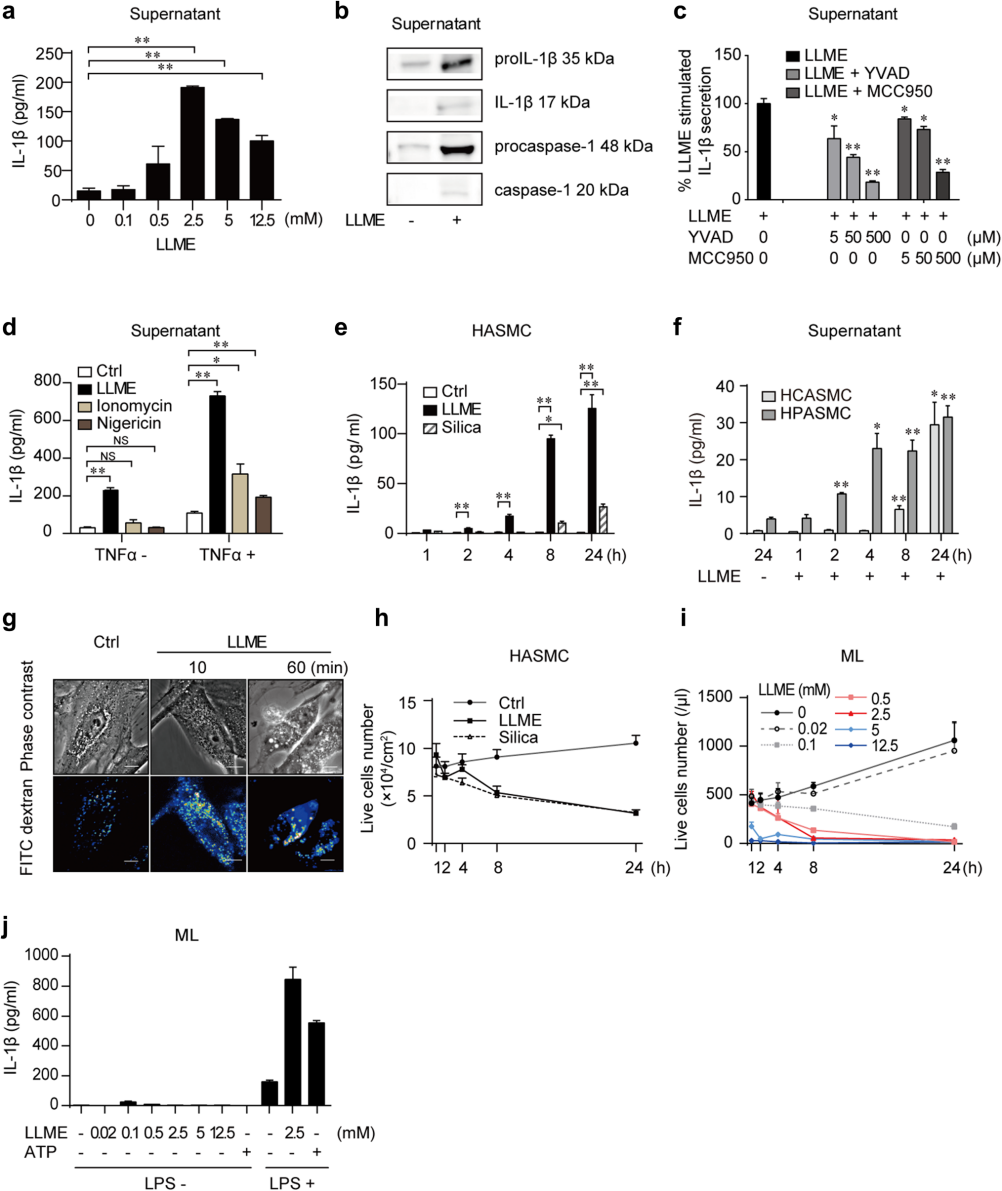
Single LMP stimulation causes the secretion of mature IL-1β from unprimed human VSMCs. **a** HASMCs were treated with LLME (0-12.5 mM), and the IL-1β concentrations were measured in the supernatant by LEGENDplex Multi-Analyte Flow Assay Kit after 24 h incubation. **p < 0.005 versus unstimulated cells. **b** Western blot of IL-1β, pro-IL-1β, caspase-1, and procaspase-1 in supernatant derived from HASMCs stimulated with LLME (2.5 mM) for 24 h. **c** IL-1β concentration in supernatant. HASMCs were cultured with increasing concentrations of YVAD or MCC950 and exposed to LLME (2.5 mM) for 24 h. *p < 0.05, **p < 0.005 versus LLME-stimulated cells without YVAD (5–500 µM), or MCC950 (5–500 µM). **d** IL-1β concentration in supernatant. Unprimed or TNFα-primed HASMCs were cultured with 2.5 mM LLME, 5 µM ionomycin, or 1 µM nigericin. *p < 0.05, **p < 0.005 versus unstimulated cells in unprimed, or TNFα-primed HASMCs. **e** Time course of the level of IL-1β in supernatant from LLME (2.5 mM)- or silica (500 µg/mL)-stimulated HASMCs. **f** Time course of the level of IL-1β in supernatant from HCASMCs and HPASMCs stimulated with LLME (2.5 mM). *p < 0.05, **p < 0.005 versus unstimulated cells. **g** LMP detection by FITC dextran in cell culture. HASMCs were stained for 1 h with FITC dextran (0.5 mg/mL) and then stimulated with LLME (2.5 mM) for 10 or 60 min. Note the swelling and bursting of the punctate after LLME stimulation (FITC dextran staining). Scale bar: 10 µm. **h** Time course of the number of live HASMCs stimulated with LLME (2.5 mM) or silica (500 µg/mL). **i** Time course of the number of live MLs stimulated with LLME (0–12.5 mM). **j** IL-1β concentration in supernatant. Unprimed MLs were stimulated with LLME (0–12.5 mM) or ATP (2.5 mM) for 24 h. LPS (1 µg/mL)-primed MLs were stimulated with LLME (2.5 mM) or ATP (2.5 mM) for 4 h. **a, c**–**f, h**–**j** Results are given as mean ± SEM (*n* = 3). Statistical significance was evaluated by Student’s *t* test

As a model of LMP, we stimulated cells with a commonly used reagent, LLME. LLME is taken up by lysosomes, where it is cut by dipeptide peptidase I and destabilizes the lysosomal membrane immediately thereafter [15, 16]. To investigate the effect of LMP on hVSMCs, we first treated a hVSMC line (HASMC) in the presence of LLME and detected the secretion of IL-1β in the culture supernatant (Fig. 1a). The amount of secreted IL-1β peaked at 2.5 mM LLME (Fig. 1a). The mature and premature forms of both IL-1β and caspase-1 were detected in the culture supernatant (Fig. 1b), proving that mature IL-1β was produced and secreted. We confirmed that mature IL-1β was processed via inflammasome activation, because the amount of secreted IL-1β with LLME stimulation was significantly decreased in the presence of a caspase-1 inhibitor, YVAD, and a NLRP3 inflammasome inhibitor, MCC950 [17] (Fig. 1c). In contrast to LLME stimulation, IL-1β was not secreted when unprimed HASMCs were stimulated with well-known activation signals of the NLRP3 inflammasome such as ionomycin or nigericin [18, 19] (Fig. 1d). Both ionomycin and nigericin treatment caused IL-1β secretion from TNFα-primed HASMCs, confirming those signals indeed act as activation signals on HASMCs (Fig. 1d). At 2.5 mM LLME, the secretion of IL-1β increased over time (Fig. 1e). We tested another LMP stimulation, silica, which physically disrupts lysosomes [20, 21]. The induced IL-1β secretion from HASMCs with silica showed a similar tendency to that with LLME stimulation, although the amount of secreted IL-1b was smaller (Fig. 1e). The IL-1β secretion by LLME stimulation was reproduced in other hVSMCs (HCASMC and HPASMC), although again the amount of IL-1β secretion was smaller than that from HASMCs (Fig. 1f).

### HVSMCs are relatively resistant to LMP-induced cell death

Since LMP usually induces cell death [21], we next evaluated the cytotoxicity of LLME on HASMCs. At 2.5 mM, LLME treatment immediately caused lysosome swelling and bursting in HASMCs, indicating the occurrence of LMP (Fig. 1g) [22]. The number of live HASMCs decreased gradually after the administration of LLME or silica, but a substantial number of cells survived 24 h later nevertheless (Fig. 1h). HCASMCs and HPASMCs also survived at least partially after 24 h of LLME treatment (data not shown). To test the cytotoxicity of LLME on monocytic cells, we treated human pluripotent stem cell-derived monocytic cell lines (MLs) [13] with LLME at 2.5 mM. All MLs perished within 24 h (Fig. 1i), showing the effects of LLME-induced LMP differ with the cell type. MLs secreted little IL-1β in response to LLME treatment, while LPS priming resulted in robust IL-1β secretion upon secondary LLME or ATP stimulation (Fig. 1j) [13], confirming that LLME acts as an activating signal for monocytic cells. Since the MLs were more sensitive to LLME-induced cell death than HASMCs, we treated MLs with a lower dose of LLME to see whether mild LMP with lower cytotoxicity can prime the MLs (Fig. 1i). Although 0.1 or 0.02 mM LMP caused lower ML cytotoxicity, the amount of IL-1β secreted from these cells was negligible when compared with the LPS-primed condition (Fig. 1j). These data support our hypothesis that LMP has cell-type-specific effects on IL-1β secretion. Overall, LMP caused the secretion of mature IL-1β from unprimed hVSMCs, suggesting that LMP has both priming and activating roles on the inflammasome in unprimed hVSMCs, but not in unprimed monocytic cells in which LLME has a role of inducing cell death. The different response in different cell types to LLME could explain the different survival rates and may be associated with LLME-induced IL-1β secretion in hVSMCs. Indeed, IL-1β secretion from LLME-treated HASMCs could be detected from 8 h after stimulation and increased with time (Fig. 1e).

### LLME-induced LMP primes the NLRP3 inflammasome of HASMCs

**Fig. 2:**
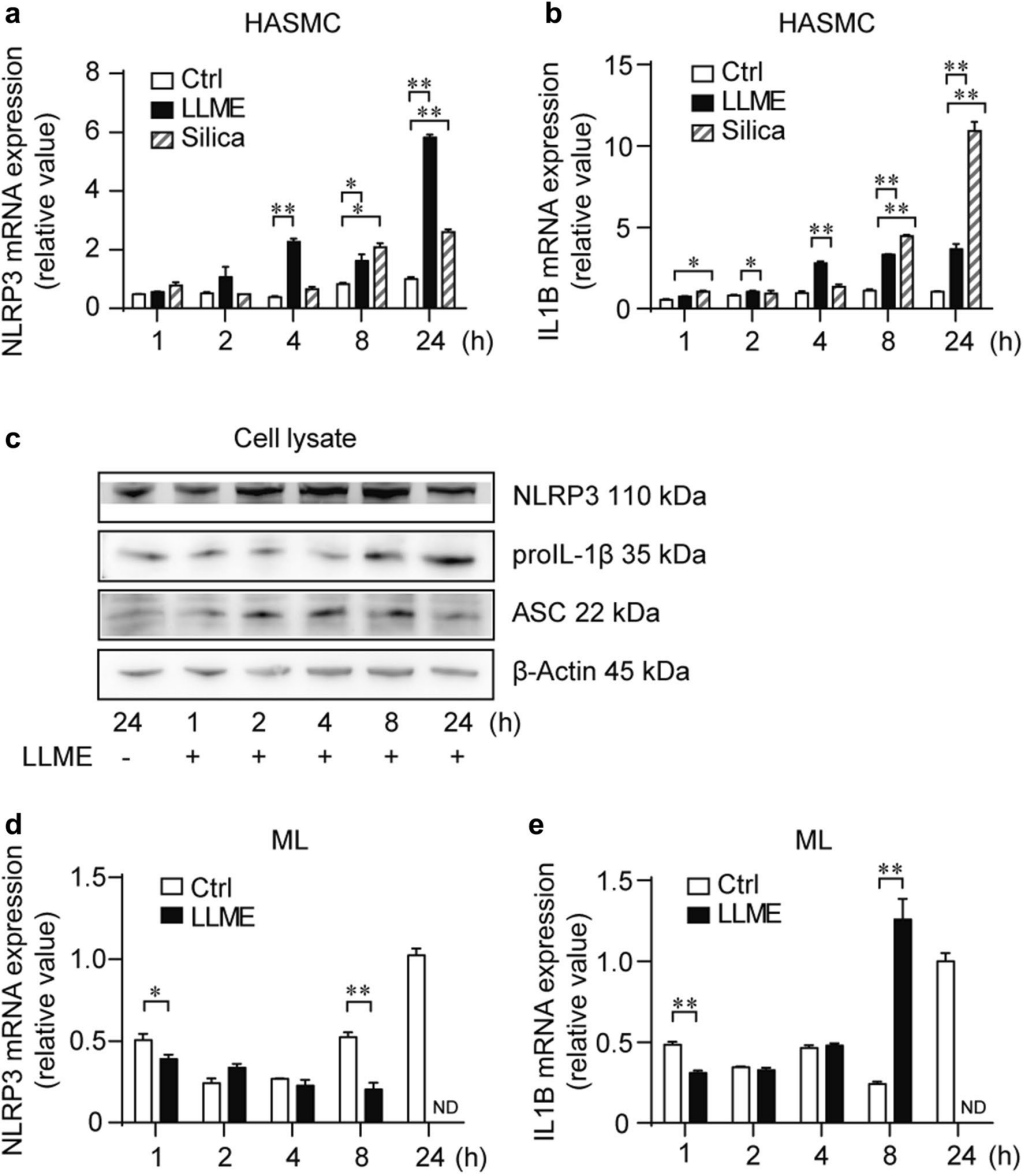
LMP primes the NLRP3 inflammasome of HASMCs. **a, b** NLRP3 and IL1B mRNA expression in HASMCs stimulated with LLME (2.5 mM) or silica (500 µg/mL) for 1–24 h. Values are relative to unstimulated cells at 24 h. **c** Western blot of NLRP3, pro-IL-1β, ASC, and β-actin in cell lysate derived from HASMCs stimulated with LLME (2.5 mM) for the indicated times. β-Actin was re-blotted from the stripped membrane for NLRP3 and proIL-β. The blot of ASC was derived from the same samples. **d, e** NLRP3 and IL-1β mRNA expression in MLs stimulated with LLME (2.5 mM) for 1–24 h. All MLs stimulated with LLME were dead at 24 h. Results are given as means ± SEM (*n* = 3). *p < 0.05, **p < 0.005 versus control cells at each time period, statistical significance was evaluated by Student’s *t* test

To confirm the priming role of LLME on HASMCs, we next examined the expression of NLRP3 and proIL-1β after LLME stimulation. The time-dependent upregulation of NLRP3 and IL1B by LLME or silica stimulation was confirmed by mRNA levels (Fig. 2a, b). The upregulation of NLRP3 and proIL-1β was also confirmed at the protein level (Fig. 2c), confirming the priming role of LLME on HASMCs. The upregulation of NLRP3 and IL1B at the mRNA level by LLME was not observed in MLs (Fig. 2d, e). Therefore, we concluded that LLME has no priming effect on monocytic cells, which is consistent with previous findings [12]. These data support a specific role of LMP on HASMCs for promoting the priming of the NLRP3 inflammasome, thereby evoking a proinflammatory response in these cells.

### LLME treatment activates canonical NF-κB pathway in HASMCs

**Fig. 3:**
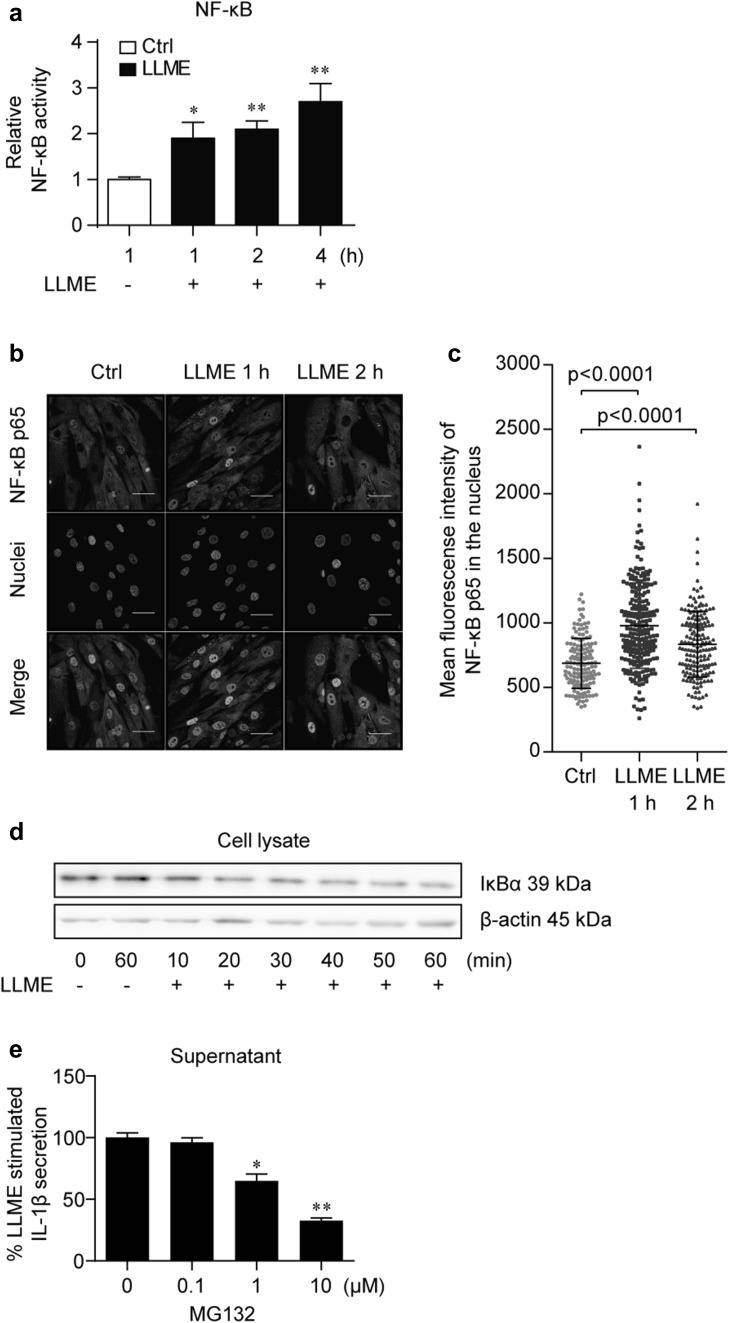
LLME treatment activates the canonical NF-κB pathway in HASMCs. **a** NF-κB activation was evaluated using an NF-κB luciferase reporter system. Cells were stimulated with or without 2.5 mM LLME for the indicated times. The relative fold change from the unstimulated condition is shown. Results are given as mean ± SEM (*n* = 6). *p < 0.05, **p < 0.005 versus unstimulated cells, Student’s *t* test. **b** Representative confocal images of immunofluorescence staining of NF-κB p65 (green). Cells were stimulated with LLME (2.5 mM) for 1 or 2 h. Cells were stained with DAPI for visualization of the nucleus (white). Scale bars: 40 µm. **c** Quantitation of the nuclear translocation of NF-κB p65 by immunofluorescence staining. The mean fluorescence intensity of NF-κB p65 merged with the nucleus in each cell is plotted. Bars indicate mean ± SD (*n* = 143–231). Mann–Whitney *U* test was used for the statistical analysis. **d** IκBα protein was analyzed after 0–60 min LLME stimulation. **e** IL-1β concentration in supernatant from LLME-stimulated HASMCs with MG132 (0–10 µM). Results are given as mean ± SEM (*n* = 3). *p < 0.05, **p < 0.005 versus LLME-stimulated cells without MG132, Student *t* test. (Color figure online)

Priming of the NLRP3 inflammasome requires the activation of intracellular signaling cascades, with one of the most important being NF-κB signaling [1, 23]. Genes involved in the inflammasome-IL-1 pathway, such as IL1B and NLRP3, are transcribed as a result of NF-κB activation [4, 24]. We hypothesized that in hVSMCs, LMP activates the NF-κB pathway, leading the cells to be primed while escaping from death. Indeed, LLME treatment increased NF-κB reporter activity in HASMCs (Fig. 3a). In line with this finding, NF-κB p65 subunit translocated to the nucleus after LLME stimulation (Fig. 3b, c). Degradation of IκBα, an essential step for the nuclear translocation of NF-κB p50/p65 subunits, was confirmed upon LLME stimulation (Fig. 3d). Furthermore, LLME-induced IL-1β secretion was inhibited by MG-132, an inhibitor of IκBα degradation (Fig. 3e), confirming that activation of the NF-κB pathway is an upstream event for IL-1β secretion. Overall, LLME treatment activates the canonical NF-κB pathway in HASMCs, and thus can act as a priming signal in these cells.

### Cathepsin B is involved in LMP-induced priming of HASMCs

**Fig. 4:**
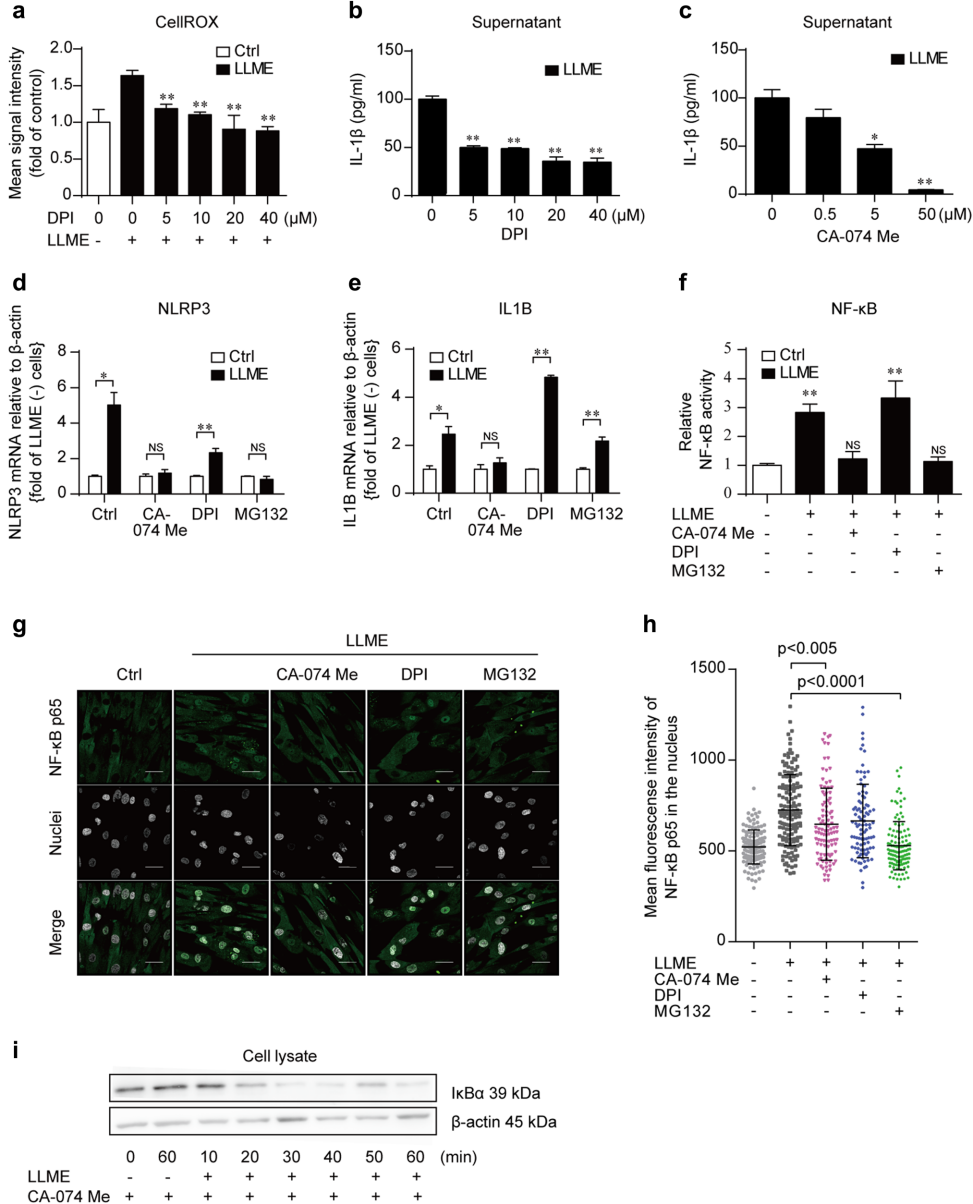
Cathepsin B is involved in LMP-induced priming of HASMCs. **a** ROS detection by CellROX in cell culture. HASMCs were stained with CellROX and stimulated with LLME (2.5 mM) and DPI (0–40 µM) for 1 h. Results are given as means ± SEM (*n* = 3). **p < 0.005 versus LLME-stimulated cells without DPI, Student’s *t* test. **b, c** IL-1β concentration in supernatant. HASMCs were cultured with increasing concentrations of DPI (B) or CA-074 Me and exposed to LLME (2.5 mM) for 24 h. Results are given as means ± SEM (*n* = 3). *p < 0.05, **p < 0.005 versus LLME-stimulated cells without DPI or CA-074 Me, Student *t* test. **d, e** NLRP3 and IL1B mRNA expression in HASMCs stimulated with or without LLME (2.5 mM) in the presence or absence of CA-074-Me (50 µM), DPI (20 µM) or MG132 (10 µM). The relative fold change from unstimulated cells in each condition is shown. Results are given as mean ± SEM (*n* = 3). *p < 0.05, **p < 0.005 versus control cells, Student’s *t* test. **f** NF-κB activation was evaluated using an NF-κB luciferase reporter system. HASMCs were stimulated with LLME (2.5 mM) in the presence or absence of CA-074-Me (50 µM), DPI (20 µM) and MG132 (10 µM) for 2 h. The relative fold change from the control cells is shown. Results are given as mean ± SEM (*n* = 9). *p < 0.05, **p < 0.005 versus control cells, Student’s *t* test. **g** Evaluation of the nuclear translocation of NF-κB p65. Representative confocal images of the immunofluorescence staining of NF-κB p65 (green). Cells were treated with LLME (2.5 mM) for 1 h in the presence or absence of CA-074-Me (50 µM), DPI (20 µM) and MG132 (10 µM). Cells were stained with DAPI for visualization of the nucleus (white). Scale bar, 40 µm. **h** Quantitation of the nuclear translocation of NF-κB p65 by immunofluorescence staining. The mean fluorescence intensity of NF-κB p65 merged with the nucleus in each condition cell is plotted. Bars indicate means ± SD (*n* = 93–145). A Mann–Whitney *U* test was used for the statistical analysis. **i** IκBα protein was analyzed after 0–60 min LLME (2.5 mM) stimulation. HASMCs were pretreated with CA-074Me (50 µM) for 1 h. (Color figure online)

LMP causes the leakage of various lysosomal content such as reactive oxygen species (ROS), cathepsin, and hydrolases into the cytoplasm [21]. Since elevated cathepsin B (CTSB) and ROS in the cytoplasm are well-known activation signals of the NLRP3 inflammasome [5, 25], we next investigated their effect on HASMCs. Cytoplasmic ROS detected by CellROX increased upon treatment with LLME in HASMCs, but decreased with treatment of an NADPH oxidase inhibitor, DPI, in a dose-dependent manner (Fig. 4a). Furthermore, DPI treatment decreased LLME-induced IL-1β secretion from HASMCs (Fig. 4b). CA-074 Me, an inhibitor of CTSB, also decreased LLME-induced IL-1β secretion in a dose-dependent manner (Fig. 4c). To discriminate the priming and activating roles of these signals on IL-1β secretion, we next investigated whether ROS and CTSB contribute to the priming of HASMCs. Pretreatment of HASMCs with DPI had minimal effect on the LLME-induced upregulation of NLRP3 and IL1B, although we could not evaluate mRNA expression beyond 4 h due to the cytotoxicity of DPI (Fig. 4d, e). On the other hand, CA-074 Me almost completely inhibited the LLME-induced upregulation of these genes (Fig. 4d, e). In line with these findings, CA-074 Me inhibited the increased LLME-induced NF-κB reporter activity and nuclear translocation of NF-κB p65 subunit, while DPI failed to inhibit either event (Fig. 4f–h). However, interestingly, CA-074 Me did not inhibit IκBα degradation (Fig. 4i), indicating an additional role of CTSB on NF-kB activation beyond the degradation of IkBa. Overall, the priming effect of LLME on HASMCs is mediated by CSTB activity, at least in part, and intracellular ROS basically contributes as an activating signal.

## Discussion

Here, we showed LMP is a sufficient signal for the secretion of IL-1β from hVSMCs. Oxidative low-density lipoproteins can induce LMP and enhance the production of IL-1β from HASMCs [26]. However, they also transduce intracellular signaling through the surface receptor CD36 [27, 28]. We, therefore, used LLME to induce LMP and found that LLME stimulation acts as a priming signal for hVSMCs along with its well-known role as an activating signal. These findings revealed a novel proinflammatory role of LMP on hVSMCs.

While the potential effects of alarmins, such as HMGB1 or IL-1α, on the priming vascular smooth muscle cells cannot be dismissed. However, we observed activation of the NF-kB signaling pathway immediately after the occurrence of LMP and the upregulation of IL1B/NLRP3 mRNA at 4 h. Considering that most of the cells were still alive at these time points (Fig. 1h), the effects were likely negligible.

LMP stimulates cellular inflammation, but also cell death [21, 29]. Indeed, in our experiments, almost all MLs died after LLME treatment. However, HASMCs were relatively resistant to LLME-induced cell death, and the surviving cells upregulated IL1B and secreted IL-1β in a time-dependent manner. Inflammatory signals such as IL-1β enhance the migration and proliferation of VSMCs. For example, transgenic mice lacking functional IL-1 do not show neointimal hyperplasia, which is induced in wild-type mice by low levels of shear stress [30]. Inflammation, such as NF-κB activity and IL-1 secretion, regulates the proliferation of VSMCs [13, 31]. Therefore, the proinflammatory response itself may be beneficial to hVSMCs for survival against fatal lysosomal disintegration. Activation of the NF-κB pathway can also contribute to cell survival [32, 33].

Furthermore, our study revealed a previously unknown role of LMP to activate the canonical NF-κB pathway in HASMCs. The enzymatic activity of CTSB seems to be associated with this event, as its inhibition decreased both NF-κB activity and IL-1β secretion. In human and mouse hepatocytes, CTSB degrades sirtuin-1, thereby positively regulating the NF-κB pathway [34]. In macrophages, CTSB supports the translation of proIL-1β [35]. These studies indicate the positive relationship between CTSB activity and the proinflammatory response to inflammatory stimuli such as LPS or TNFα. Interestingly, in murine macrophages, LLME induces the degradation of intracytoplasmic IL-1β, contrary to our results [12]. Thus, the precise molecular mechanism that connects CTSB and NF-κB activation in HAMSCs remains to be elucidated.

Classically, the infiltration of hematopoietic cells initiates vascular inflammation. Activated monocytes and macrophages migrate into subintimal spaces and release various cytokines and chemokines including IL-1β [36, 37]. Consistent with this theory, most IL-1β expression is observed in macrophages and endothelial cells in the pathological specimen, but some VSMCs also express IL-1β [11, 37]. In previous reports, the role of IL-1β in VSMCs has been relatively unattended. Our data support a novel role of LMP as a single stimulus for the secretion of IL-1β from hVSMCs even in the absence of innate immune cells. This characteristic feature of hVSMCs suggests that spontaneous inflammation occurs in vascular media before the infiltration of immune cells or the dysfunction of vascular endothelial cells. Further studies using in vivo models are required to validate this hypothesis. To conclude, hVSMCs may be an important initiator of the sterile inflammatory response caused by lysosomal disintegration.

## Abbreviations

PRP: Pattern recognition receptors
ASC: Apoptosis-associated speck-like protein
ROS: Reactive oxygen species
LMP: Lysosomal membrane permeabilization
hVSMC: Human vascular smooth muscle cell
HASMC: Human aortic smooth muscle cell
HCASMC: Human coronary artery smooth muscle cell
HPASMC: Human pulmonary artery smooth muscle cell
LLME: Leu-Leu-*O*-methyl ester
YVAD: Ac-Tyr-Val-Ala-Asp-CHO
DPI: Diphenyliodonium chloride
ML: Monocytic cell lines
qPCR: Quantitative polymerase chain reaction
CTSB: Cathepsin B
